# Melancholic versus non-melancholic depression: differences on cognitive function. A longitudinal study protocol

**DOI:** 10.1186/1471-244X-10-48

**Published:** 2010-06-17

**Authors:** Saray Monzón, Margalida Gili, Margalida Vives, Maria Jesus Serrano, Natalia Bauza, Rosa Molina, Mauro García-Toro, Joan Salvà, Joan Llobera, Miquel Roca

**Affiliations:** 1 Institut Universitari d'Investigacions en Ciències de la Salut (IUNICS), University of Balearic Islands (UIB), Ctra, Valldemossa km 7.5, 07122 Palma de Mallorca, Balearic Islands, Spain; 2 Unitat de Psiquiatria i Psicologia Clinica, Hospital Joan March, University of Balearic Islands, Ctra, Sóller s/n, 07110, Bunyola, Balearic Islands, Spain; 3 Servei de Psiquiatria, Hospital de Manacor, Ctra, Manacor-Alcudia s/n, 07500 Manacor, Balearic Islands, Spain; 4 Direcció General d'Avaluació i Acreditació, Conselleria Salut i Consum, Govern de les Illes Balears, C/de Carles I, 6, 07003 Palma de Mallorca, Balearic Islands, Spain

## Abstract

**Background:**

Cognitive dysfunction is common among depressed patients. However, the pattern and magnitude of impairment during episodes of major depressive disorder (MDD) through to clinical remission remains unclear. Heterogeneity of depressive patients and the lack of longitudinal studies may account for contradictory results in previous research.

**Methods/Design:**

This longitudinal study will analyze cognitive differences between CORE-defined melancholic depressed patients (n = 60) and non-melancholic depressed patients (n = 60). A comprehensive clinical and cognitive assessment will be performed at admission and after 6 months. Cognitive dysfunction in both groups will be longitudinally compared, and the persistence of cognitive impairment after clinical remission will be determined.

**Discussion:**

The study of neuropsychological dysfunction and the cognitive changes through the different phases of depression arise a wide variety of difficulties. Several confounding variables must be controlled to determine if the presence of depression could be considered the only factor accounting for group differences.

## Background

Over the last years cognitive dysfunction has increasingly been recognized as a core feature of major depressive disorder (MDD). Clinical studies have focused on the pattern and magnitude of impairment during and between episodes of MDD as well as the neuropsychological domains affected and the origin of these abnormalities [1]. However, results from neuropsychological and neuroimaging studies are still controversial. These contradictory results could be explained mainly by two methodological factors. The first factor is the absence of homogeneity in clinical samples. The heterogeneity of patients evaluated in clinical studies may derive from the different criteria (DSM, ICD) currently used to diagnose MDD and its subtypes. Some authors have pointed out that these criteria poorly identify samples for clinical and outcome studies [2,3]. The second factor explaining controversial results is related to the lack of longitudinal studies that focus on the changes on cognitive function produced through the clinical course of depression. Whether cognitive impairment manifested during periods of depression is long lasting or improves after remission and recovery remains a central issue of study [4]. The cognitive domains affected have neither been clearly identified [5].

To overcome the limitation derived from the first methodological factor, a more acurate selection of depressed patients is required. A pattern of cognitive dysfunction may be more evident in a form of depression characterized by biological markers than in more heterogeneous depressed samples. Melancholia is a disorder with definable clinical signs that identifies more specific populations than the DSM-IV [3]. It describes episodes in which physical symptoms are predominant and is opposed to a reactive or non-melancholic form of depression in which the presence of low mood and tearfulness is frequent and biological markers are not predominant [6].

Overcoming the limitation derived from the lack of longitudinal data imply the development of cohort studies. Longitudinal assessment of cognitive functions seems to be a potentially powerful method of identifying and distinguishing state-related from trait-related cognitive deficits [4]. Previous studies report residual neuropsychological deficits in melancholic patients despite improvement in their depressive symptomatology [7]. Particularly, persisting executive functions and memory disturbances have been observed. This would indicate that some cognitive dysfunction may not be simply secondary to mood disturbances in depression but may represent trait vulnerability markers for MDD. Deficits in other domains of cognitive performance appear to be more state-dependent [8]. The high risk of relapse in depression makes it important to analyze the existence of persisting cognitive impairments during remission, recovery and the euthymic phase of depression. A better understanding of these issues is crucial as it has been suggested that cognitive impairment worsens for every episode of depression and that the observed cognitive impairment in a nonsymptomatic phase of depression may be related to the number of previous episodes [9].

The course of cognitive changes through to clinical improvement in samples of depressed participants with and without melancholia has scarcely been longitudinally studied [6]. The present study aims to analyze longitudinally the cognitive performance of a homogeneous sample of depressed patients. Results of this study can have relevant implications for treatment and neuropsychological rehabilitation.

## Methods/Design

### Objectives

The general aim of this study is to analyze cognitive differences between melancholic depression (MD) and non-melancholic depression (NMD), and compare longitudinally the cognitive dysfunction in both types of patients, determining whether these alterations remain after clinical remission. This will enable to define a specific cognitive dysfunction pattern depending on these subtypes of depression. Finally, the study will analyze the correlation between the severity of these dysfunctions and the number of previous depressive episodes.

Our hypotheses are that specific cognitive deficits during acute phase of MDD will be different between patients with MD and NMD. Melancholic depressed patients are expected to show a poorer performance on memory, attention and executive function tasks at baseline. We hypothesize that cognitive deficits in the group with melancholia will persist at the follow-up assessment despite the presence or absence of clinical remission. Cognitive deficits in the non-melancholic depression group will only be expected to persist in those patients who fail to achieve clinical remission at the 6-month follow-up assessment while is presumed to be reduced or to disappear in those who have achieved it. Finally, the number of previous episodes of depression of melancholic patients is predicted to positively correlate with cognitive deficits severity.

### Design

This is a multicenter, observational longitudinal cohort study that will include 120 outpatients treated for an acute episode of MDD. 60 patients with melancholic depression will be compared to 60 non-melancholic depressed patients matched by age, gender and education level. All patients will be included during an acute phase of the disorder and will undertake standard psychopharmacological treatment for depression.

Information regarding socio-demographic and clinical features will be collected at the baseline evaluation. A neuropsychological protocol will be administrated both at the baseline and the six-month follow-up assessments. The obtained results will be compared between four groups: melancholic patients showing clinical remission, melancholic patients who fail to achieve clinical remission, non-melancholic patients showing clinical remission and, finally, non-melancholic patients who fail to achieve clinical remission.

In our study clinical remission will be operatively conceptualized as having a HDRS total score ≤ 7 both at the 6-month follow-up assessment and in an additional interview that will be performed two weeks later.

This study will be performed in accordance with the Helsinki Declaration of the World Medical Association Assembly and the Madrid Declaration of the World Psychiatric Association (WPA). The study protocol has received the approval of the Ethics and Clinical Research Committee of the Balearic Islands (Palma de Mallorca, Spain).

### Setting

All patients will be identified and recruited from different centers across the island of Majorca (Spain), including 4 Outpatient Psychiatric Units (Joan March Hospital, Son Dureta Hospital, Son Llàtzer Hospital and Hospital of Manacor), 2 Mental Health Units and 9 Primary Care Centers.

### Measuraments

1. ***Sociodemographic and clinical questionnaire ***designed specifically for this study. Relevant sociodemographic information regarding gender, date of birth and age, marital status, education level and occupational status, as well as clinical data regarding age of disorder onset, number of previous episodes and current pharmacological treatment (type of agent, mean dose and time of administration) will be collected.

2. ***DSM-IV criteria for Major Depressive Disorder ***[10].

3. ***17-item Hamilton Depression Rating Scale ***(HDRS) [11]. This is the most commonly used severity scale in clinical practice and research with mood disorders [12,13].

4. ***The CORE system for melancholia ***(CORE) [14] is an 18-item scale which assesses features of melancholic depression such us retardation, agitation and non-interactivity by behavioural observation. Each sign is rated on a 4-point scales (0-3) by clinicians or a trained observer. The CORE distinguish melancholia from other residual depressive disorders. Depressed patients will be allocated to the CORE-defined melancholic group if they store 8 or more. The Spanish version of this scale is currently under validation. Our group is involved in this process.

5. ***The Mini-Mental State Examination ***(MMSE) [15] has been widely used as a screening tool both in clinical practice and in research. The MMSE total score is widely accepted as an indicator of the severity of cognitive impairment. It is highly sensitive (87%), and highly specific (82%) in the detection of dementia. The success of the MMSE relay on the fact that it can be admistered in a very short time. It is able to objectify cognitive status as a single global score and to track decline from mild to severe dementia.

6. ***The Clinical Global Impression rating scales ***(CGI-S, CGI-I) [16] are commonly used measures of symptom severity, treatment response and efficacy of treatments in treatment studies of patients with mental disorders. The Clinical Global Impression - Severity scale (CGI-S) is a 7-point scale that requires the clinician to rate the severity of the patient's illness at the time of assessment, relative to the clinician's past experience with patients who have the same diagnosis. The Clinical Global Impression - Improvement scale (CGI-I) is a 7 point scale that requires the clinician to assess how much the patient's illness has improved or worsened relative to a baseline state.

Cognitive assessment, administered in this fixed order:

1. ***Trail Making Test-Parts A and B ***[17] In this test the subject consecutively connects numbered circles (Trails A) and then connects numbers and letters in alternating sequence (Trails B). Both are timed tests of visuomotor speed, with Trails B also being a test of set-switching. A high score indicates poor performance. Results from a recent study [18] suggest that TMT-A requires mainly visuoperceptual abilities whilst TMT-B reflects primarly working memory and secondarily task-switching ability. The difference score B-A would minimize visuoperceptual and working memory demands, providing a relatively pure indicator of executive control abilities.

2. ***Digit Span subtest of the Weschler Adult Intelligence Scale, 3rd edition ***(WAIS-III) [19]. Digit span forwards is used as a measure of attention and phonological storage in working memory whilst digit span backwards also draws on mental flexibility and is additionally regarded as a measure of executive function [20].

3. ***Stroop Colour Word Test ***(SCWT) [21]. This task measures selective attention, freedom from distractibility and response inhibition [20]. Three 45-second trials are used in this test where the subject is asked to read out three colour names printed in black ink as fast as posible. The subject then is presented with the same words printed in a colour different from the color which it names. The subject is instructed to name, as fast as posible, the colour of the ink in which the word is printed. The time taken to complete the task increases significantly under these conditions and thi is called the 'interference effect'. The interference score is calculated by substracting Trial 1 (naming words) from Trial 3 (stating the colour in which the word is printed), a higher score indicating greater interference.

4. ***Tower of London, 2nd Edition ***(TOL-DX) [22]. This is a test of planning that taxes central executive function. Subjects rearrange a set of spheres to match a given target arrangement in a specified number of moves. Accuracy and latency are recorded [23].

5. ***The Controlled Verbal Fluency Task ***(FAS) [24]. This is a timed test in which the subject is asked to generate words starting with letters F, A and S as fast as posible. This task involves development of a strategy to produce the words and is dependent on psychomotor speed. A standard score of 12 words beginning with a specific letter is expected to be produced within 1 minute.

6. ***Semantic Verbal Fluency ***(ANIMALS). This test requires the generation of words corresponding to a specific semantic category. The number of correct words in one minute is counted. A mean of 16 words is expected to be produced as a standard score [25].

7. ***Finger Tapping Test ***(FTT) [26]. Originally developed as part of the Halstead Reitan Battery (HRB) of neuropsychological tests, the Finger Tapping Test is a simple measure of motor speed and motor control. The speed, coordination and pacing requirements of finger tapping can be affected by levels of alertness, impaired ability to focus attention, or slowing of responses.

### Study sample

The recruitment strategy will be performed by General Practicioners and Psychiatrists involved in the study. Any outpatient who might fulfil all the inclusion criteria will be offered to participate in the study. Male and female outpatients who are eligible and sign the written informed consent will be entered into the study.

At the screening interview the Hamilton Depression Rating Scale (HDRS) will be used to determine depression severity while the CORE Scale [27] will allow distinguishing melancholic depressive patients (MD) from non-melancholic depressive patients (NMD). Inclusion criteria are: a diagnosis of DSM-IV unipolar MDD, to be under treatment with antidepressive agents or to initiate this treatment, age between 18-55 years, a HDRS greater than or equal to 18, a total score greater or equal to 8 in the CORE scale for the Melancholic Depression patients and under 8 for the non-melancholic depression individuals and enough capacity for understanding and signing the written informed consent form. Exclusion criteria are: history of medical conditions that can entail cognitive deterioration, history of head injury or neurological disorder, current psychotic symptoms, current treatment with antipsychotic or mood stabilizer agents, electroconvulsive therapy in the 6 months prior to the study; a diagnosis of mental retardation, and disability to understand and complete the cognitive assessment.

### Procedure

Clinical interview will be performed both at baseline and follow-up assessments. The structured clinical interview will address sociodemographic characteristics, medical history and current medication prescription. Clinical scales and questionnaires will also be administrated. The cognitive assessment will follow the clinical interview. It will also be conducted at baseline and 6 months later. The follow-up assessment will take place after 6 month of inclusion in the study independently of the patient clinical state (acute phase, clinical remission or recovery). All patients showing a HDRS total score ≤ 7 in the follow-up assessment will be re-interviewed after two weeks. They will be considered as being in remission of depressive symptoms if the HDRS total score at this time remains equal or under 7 points. All assessments will be performed by two trained psychologists (SM, MV), and will be carried out in the morning in order to avoid the confounding effects of diurnal fluctuation in mood and cortisol levels which have been pointed out to influence cognitive performance [28].

### Description of all comparisons

Obtained data regarding sociodemographic and clinical features of the depresive episode at baseline assessment will be compared between melancholic and non-melancholic depressed patients in order to detect any significant difference.

Intergroup analysis will provide information regarding test score differences between melancholic and non-melancholic depressed patients both at baseline and the follow-up assesments. Furthermore, comparisons between melancholic and non-melancholic depressed patients showing clinical remission will be carried out. Melancholic and non-melancholic depressed patients who fail to achieve clinical remission will be compared as well.

Intragroup comparisons will provide information regarding test score differences between the baseline and the follow-up assessment for each group. Thus, the cognitive profile of melancholic depressed patients at baseline will be compared to the cognitive profile obtained at follow-up assessment and the cognitive profile of non-melancholic patients will be compared likewise. Test scores obtained by melancholic depressed patients who show clinical remission will be compared at both times of assessment. Test scores of non-melancholic depressed patients showing clinical remission will be compared likewise. The same comparisons will be undertaken with melancholic and non-melancholic depressed patients who fail to achieve clinical remission.

### Statistical Analyses

Obtained data will be analyzed with the Statistical Package for the Social Sciences (SPSS) version 17. Univariate descriptive analysis of the included variables will be performed. For metric variables, central tendency measures, measures of statistical dispersion and measures of position will be applied. In the case of categorical variables, univariate description will be obtained with relative frequencies. Bivariate analysis will be performed by means of the Student's t-test for the comparison of two means, the Analysis of Variance (ANOVA) for the mean comparison of more than two groups, the Mann-Whitney test for ordinal variables and the Chi-square test for categorical variables. Multivariate analysis will be calculated with multiple linear regression and logistic regression models.

### Type of analysis

The sample size was calculated assuming an alpha risk of 0.05 and accepting a beta error rate of 20%, which corresponds to a study power of 80%. Expecting a 20% loss, the necessary sample size will be 60 patients in each group, constituting thus a total sample of 120 patients.

## Discussion

To our knowledge, this will be the first study design that longitudinally compares cognitive functioning in depressed patients with and without CORE-defined melancholic features. The imprecision of depressive symptoms limit the capacity of any measure to delineate and measure melancholia [29]. Nowadays, there is no a gold standard measure of melancholia. However, while other measures such as the Newcastle and the Hamilton Depression Rating Scale essentially generate depression severity scores, the CORE scores seems to be sufficient to the clinical definition of melancholia [2].

In the present study, the cognitive profile of those patients showing clinical remission of depressive symptoms will be separately analyzed from the cognitive profile of those patients who fail to achieve remission of symptoms. The design seeks to overcome two important methodological limitations of previous investigation: the heterogeneity of depressed patients samples and the lack of longitudinal studies focusing on cognitive functioning changes from the acute phase of depression to clinical remission.

The study of neuropsychological dysfunction and the cognitive changes through the different phases of depression arise a wide variety of problems. Some of them are closely related to the nature and intrinsic characteristics of neuropsychological assessment and the interpretation of results. Most of the neuropsychological tests that will be used in our study were primarily developed for brain-damaged patient examination. As highlighted by Austin et al. [30], the application of this tests to patients with functional psychiatric disorders calls for caution in interpreting test results. It is also important to recognize that test complexity adds more difficulty to the interpretation of findings. Many neuropsychological tests involve a number of complex cognitive processes. Few (if any) measure only one circumscribed cognitive function and it has been suggested that assigning tests to specific cognitive categories is somewhat arbitrary [1].

Another major difficulty is related to the wide variety of confounding variables that must be controlled in neuropsychological testing. The presence of depression should be the only difference likely to impact on cognitive function [1]. Difference in neuropsychological function not attributable to depression could be explained by differences between groups in variables such as gender, age, severity of depression, antidepressant treatment type and number of previous episodes, among others.

### Gender

Few studies of neuropsychological impairment in MDD have considered the effects of gender on cognitive performance [1]. The present study will apply no restrictions regarding gender for inclusion, although according to some authors, there would be a gender-related specificity, since depressed women appear to perform significantly worse as compared with depressed men [31]. However, gender will be taken into account as a covariate in statistical analysis.

### Age

Age is associated with a progressive decline in cognitive function. Difficulties produced by age (such as mental inflexibility, higher susceptibility to distractors and perseveration) [32] involve the same specific domains that are impaired in more severe (and melancholic-type) depression. Most of the studies investigating the association between depression and cognitive dysfunction have been conducted among middle-aged and elderly patients or among patients regardless of their age [8]. In any study of cognitive deficits in depression, episodes of depression that occurs for the fist time in later life should be considered as a potential confounder. It has been demonstrated that there is a greater contribution of the vascular pathology in late-onset cases of depression [33]. Although there is no consensus regarding the age of onset cut-off for late-onset depression [34], it is generally established as later than age 60. Therefore, in our study a cut-off age of 55 years was established in order to clearly exclude possible cases of late-onset depression and to avoid any other significant age-related confounders.

### Severity of depression

Many studies have investigated the effect of severity of depression on neurocognitive task performance. Overall, results suggest the importance of applying corrections for depression severity when comparing patients with different subtypes of MDD, as it seems quite clear that differences in mean depression scores of patient samples could account in part for the different levels and patterns of cognitive impairment observed [35]. In fact, there are no studies investigating the relationship between melancholia and neuropsychological impairment showing a definitively different pattern unrelated to measures of severity.

### Antidepressant treatment type

The use of medication during neuropsychological assessment is an important confounding variable. It is therefore surprising that most of the works studying the persistence of cognitive disturbances beyond the symptomatic phase of MDD have overlooked the possible impact of medication on cognitive function [36]. Antidepressant (especially tricyclic antidepressant) and benzodiazepines can deteriorate performance due to their effects on cognitive and motor functions. Many antidepressants exhibit pharmacological properties that can explain why such dysfunctions should be expected. However, some works have shown that antidepressants can also improve performance, possibly due to its positive effect on mood [37]. Indeed, improvement in memory and attention skills in depressed patients treated with selective serotonine reuptake inhibitors (SSRI) such as fluoxetine or paroxetine have been described [38].

Control for the potential effects of medication will be applied in our study by establishing treatment type as a covariate in statistical analysis. Electroconvulsive therapy received in the six months preceding study admission was established as an exclusion criteria.

### Number of previous episodes

Little is known regarding the influence of age of onset, duration and number of episodes of MDD on cognitive function in depressed patients. A great part of the studies concerning number of episodes and cognitive performance in depression have mainly been related to memory rather than to attention or executive functions [4]. Some studies have pointed out that there would be a worsening on cognitive function for every episode of depression [39,40].

## C) Strenghts and limitations

Among the strengths of this study it must be highlighted the large number of participants to be included as well as the homogeneity of the sample of depressed patients, especially with respect to their melancholic or non-melancholic condition. We have applied a narrow definition of melancholia (measured by the CORE system) instead of the broad definition of the DSM which, although being 'the most frequently used' reference point, have been pointed out to have poor agreement with the core features of melancholia derived from empirical studies [41].

As noted above, only few studies have compared cognitive performance in melancholic MDD patients longitudinally. However, it has been suggested that a measure of chronic depressive symptoms taken over a period of time may share a closer relationship with cognitive function [35].

Another major strength of the present study is related to the conceptualization of remission in depression. Establishing a standarized definition of remission is a controversial issue. According to most definitions, it is described as a state of minimal to no symptoms and a return to normal functioning [42]. Most studies have usually conceptualized remission without establishing a specific duration criterion. To many authors, this seems to be an inappropriate way to operatively conceptualize remission, as longitudinal assessment is required [43]. Thus, in our study clinical remission will be conceptualized as having a HDRS total score ≤ 7 both at the 6-month follow-up assessment and at an additional assessment that will be carried out two weeks later.

The number of significant confounders in neuropsychological testing is high. However, control of a variety of confounders such as gender, age or type of treatment among others will be applied.

The present study shows some limitations as well. Although the neuropsychological assessment is comprehensive, not every cognitive function will be assessed.

Patients using other medication different to antidepressants or benzodiazepines will be excluded. So will be patients who have received electroconvulsive therapy in the six months previous to screening for this study. This could limit somehow the extent of results' applicability.

Another possible limitation is that the presence of comorbid Axis I, Axis II or Axis III DSM-IV disorders will not be taken into account. Although it is possible that in some way this could increase the cognitive impairment associated with MDD, everyday practice shows that comorbidity is not uncommon and exclusion of patients due to comorbid disorders could limit generalisability of results. All eligible patients with a history of medical conditions that could entail cognitive deterioration or with a history of head injury or neurological disorder will be excluded. However, it is possible that some of the patients that will be included in the study may present clinical symptoms of an incipient undiagnosed clinical picture. This possibility must be taken into account and calls for caution in the interpretation of results.

Findings of the present study will contribute to bring light to the melancholic subtype of depression debate. Moreover, they may help to identify differential neurocognitive profiles between those depressed patients who remit and those who do not achieve remission and would give rise to future research of specifically tailored treatments.

## Abbreviations

MDD: Major Depresive Disorder; MD: Melancholic depression; NMD: Non-melancholic depression; HDRS: Hamilton Depression Rating Scale.

## Competing interests

The authors declare that they have no competing interests.

## Authors' contributions

SM, MG, MR, MG-T and JS are the principal researchers and developed the original idea of the study. MG, RM, and JLL developed the study design and will perform the statistical analysis. SM, MV, NB and MJS will perform all the clinical and neuropsychological assessments. All authors have read and corrected draft versions of the present manuscript and approved the final version.

## Pre-publication history

The pre-publication history for this paper can be accessed here:

http://www.biomedcentral.com/1471-244X/10/48/prepub
